# Supplementary material for: A New Approach for a Safe and Reproducible Seeds Positioning for Diffusing Alpha-Emitters Radiation Therapy of Squamous Cell Skin Cancer: A Feasibility Study
Source: Cancers (Basel). 2022 Jan 4;14(1):240. doi: 10.3390/cancers14010240 (PMC8750419; doi:10.3390/cancers14010240)
Supplement: Supplementary file 1 [file cancers-14-00240-s001.zip › cancers-1494610-supplementary.pdf]

Supplementary materials: A New Approach for a Safe and Reproducible Seeds Positioning for Diffusing Alpha-Emitters Radiation Therapy of Squamous Cell Skin Cancer: A Feasibility Study

Giacomo Feliciani, Salvatore Roberto Bellia, Massimo Del Duca, Giorgio Mazzotti, Manuela Monti, Ignazio Stanganelli, Yona Keisari, Itzhak Kelson, Aron Popovtzer, Antonino Romeo and Anna Sarnelli

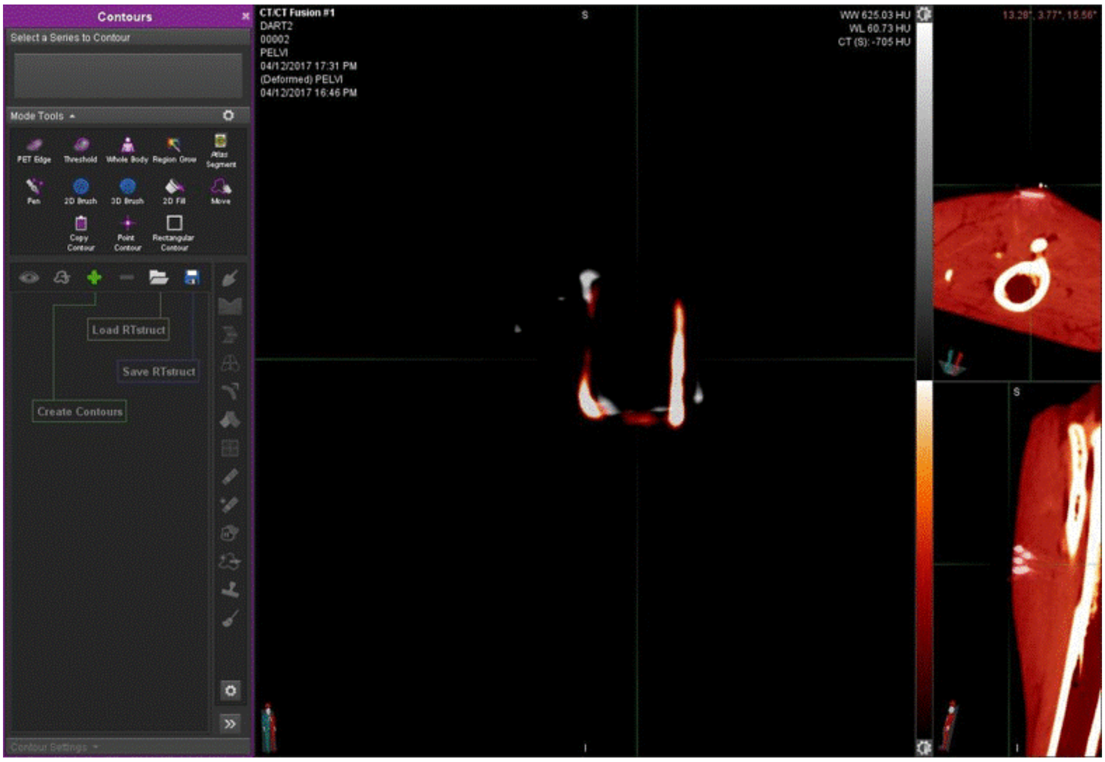

Figure S1. Animated GIF showing the rigid fusion between pre and post implant CT of the phantom in coronal view.

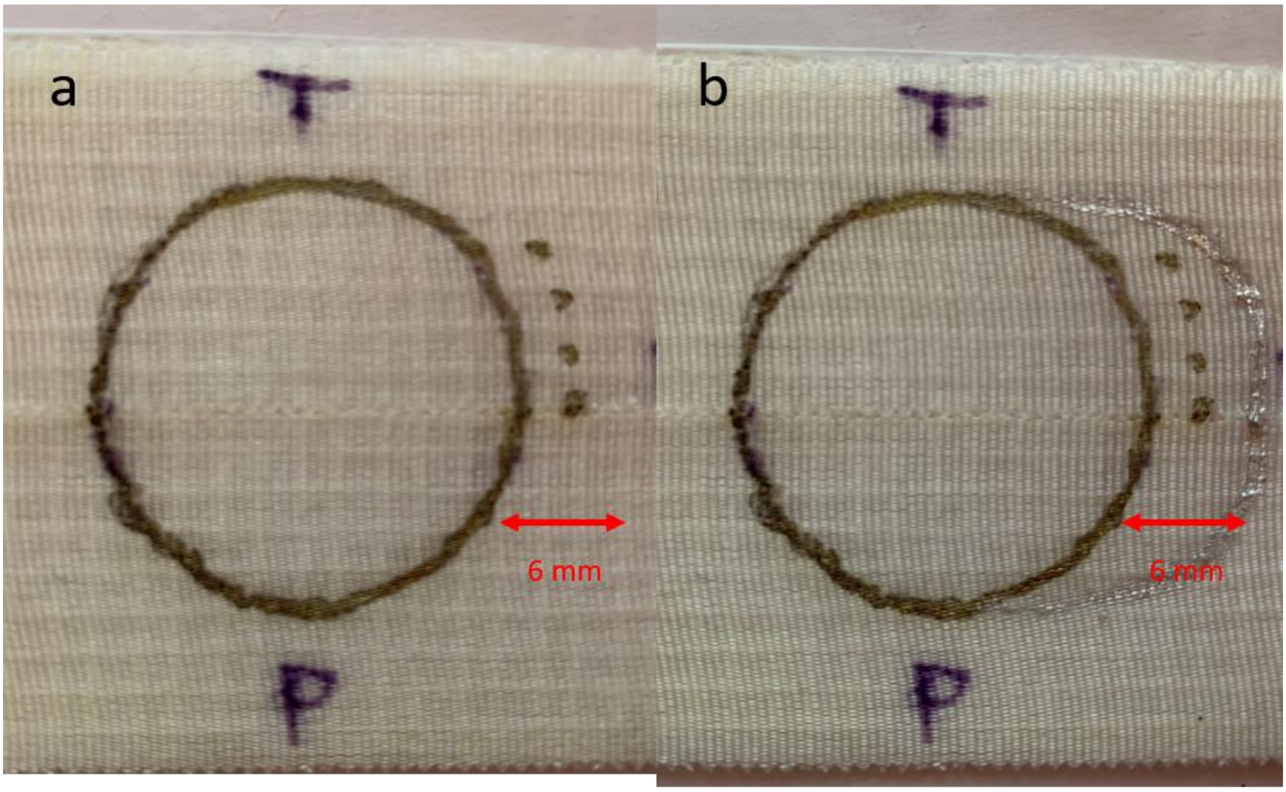

Figure S2. (**a**) CTV contouring of the visible lesion on the template mesh with silver-based ink. (**b**) Contouring update after consideration about subcutaneous invasion of the tumour based on PET/CT information.
